# Development of a Chikungunya Arthritis Disease Activity Score (CHIK-DAS) Based on a Prospective Cohort Study

**DOI:** 10.33696/immunology.6.211

**Published:** 2024

**Authors:** Aileen Y. Chang, Samuel Simmens, Hugh Watson, Richard L. Amdur, André Siqueira, Abigale Proctor, Sarah Tritsch, Carlos Andres Herrera Gomez, Liliana Encinales, Alfonso Sucerquia Hernández, Jose Forero-Mejía, Alejandro Jaller, Juan Jose Jaller, J. Kennedy Amaral, Ilana Heckler, Gary L. Simon, Larry Moreland, Andres Cadena, Gary S. Firestein

**Affiliations:** 1 Department of Medicine, George Washington University, Washington, DC, USA; 2 Department of Biostatistics and Bioinformatics, Milken Institute School of Public Health, George Washington University, Washington, DC, USA; 3 Medical Development and Translational Science Department, Evotec ID, Lyon, France; 4 Department of Hepatology and Gastroenterology, Aarhus University, Aarhus, Denmark; 5 Feinstein Institute for Medical Research, Northwell Health, Manhasset, New York, USA; 6 Instituto Nacional de Infectologia Evandro Chagas – Fiocruz Lab. Doenças Febris Agudas, Manguinhos, Rio de Janeiro, Brazil; 7 Department of Global Health, Milken Institute School of Public Health, George Washington University, Washington, DC, USA; 8 Department of Medicine, Allied Research Society, Barranquilla, Atlántico, Colombia; 9 CIMEDICAL, Barranquilla, Atlántico, Colombia; 10 Department of Health Sciences, Federal University of Cariri, Barbalha, Ceará, Brazil; 11 Department of Scientific Affairs, EUROIMMUN US, Mountain Lakes, New Jersey, USA; 12 Department of Medicine, University of Colorado, Aurora, Colorado, USA; 13 Centro de Investigación, Clínica de la Costa SAS, Barranquilla, Atlántico, Colombia; 14 Department of Medicine, UC San Diego School of Medicine, San Diego, CA, USA

**Keywords:** Chikungunya, Arthritis, Disease severity score, Quality of life, Disability, Pain, Clinical trials

## Abstract

**Objective:**

Chikungunya virus is spread by mosquitos and causes a debilitating chronic arthritis that has no standard treatment to date and no specific measures of disease activity. The objective of this expert group was to develop a measure of chikungunya arthritis that would be useful for clinical trials and patient care.

**Methods:**

A group of rheumatologists and biostatisticians experienced in the clinical and pathological mechanisms of chikungunya evaluated component measures for inclusion in a chikungunya arthritis disease activity score (CHIK-DAS). Utilizing data from a Colombian cohort of 158 chikungunya arthritis patients, linear regression identified components that were independently associated with patient reported outcomes assessing disability, pain, physical and mental quality of life and mobility. A preliminary instrument was developed using multiple imputation and regression backward selection. Cutoffs for grading disease severity were determined.

**Results:**

Stiffness, ankle tenderness, and a 30 tender joint count that included the 28 joints traditionally included in the Disease Activity Score-28 were selected in a regression model predicting a composite of five patient reported outcomes. A CHIK-DAS scoring formula was developed through a weighted combination of these selected variables. In comparison to the DAS-28, the CHIK-DAS had improved predictive value for a composite outcome of disability, pain, physical and mental quality of life and mobility. Disease activity cutoffs were defined for remission (<40), mild (40–49.99), moderate (50–59.99) and severe (60+) disease.

**Conclusion:**

The CHIK-DAS is a chikungunya specific measure of disease activity that includes the DAS-28 with the addition of ankle tenderness and a stiffness item that are prominent components of chikungunya arthritis. CHIK-DAS may be used as a specific measure of disease activity in chikungunya arthritis in clinical trials and patient care. This metric needs further validation in additional cohorts.

## Background and Objectives

Chikungunya virus (CHIKV) is an alphavirus spread by mosquitos, primarily *Aedes aegypti* and *Aedes albopictus*, that causes a debilitating febrile illness called chikungunya fever (CHIKF) [1]. CHIKF usually presents in two phases, starting as an acute illness, characterized by high fever, polyarthralgia and polyarthritis, headache, maculopapular skin rash, sometimes pruritic, and severe fatigue, commonly accompanied by anorexia, nausea, vomiting and diarrhea. This first phase usually resolves within 5–14 days [2,3]. The second phase is characterized by chronic arthritis > 3 months that can last for years after infection [1]. Chronic chikungunya arthritis (CCA) causes significant morbidity [4] and loss of economic productivity [4,5]. Multiple other alphaviruses such as Mayaro, Sinbis, Ross River, and O’nyong’nyong also cause severe arthritis [6]. CHIKV arthritis occurs in underserved regions and is a largely neglected disease for clinical trials. There is currently no standard evidence-based treatment for alphavirus-induced arthritis, although some clinical trials are in progress to validate potential therapies.

The arthritis caused by CHIKV is similar to rheumatoid arthritis (RA) in terms of its chronicity, disease severity and relapsing-remitting nature [1]. As such, measures of disease activity developed for RA such as the Disease Activity Score-28 [7] are commonly used to assess disease activity in chikungunya arthritis (CHIKA). However, important differences in the clinical presentation of CHIKA suggest that a specialized chikungunya disease activity score is needed to accurately measure response to therapy in clinical trials and in routine clinical practice. For example, the smaller joints of the lower extremity are more commonly affected in CCA and stiffness in the absence of swelling is associated with disability and decreased quality of life (QoL) in CHIKA [8]. Therefore, these measures were evaluated for inclusion in the targeted measure.

We now describe the development of the Chikungunya Arthritis Disease Activity Score (CHIKDAS) which is a modification of the Disease Activity Score-28 (DAS-28) for RA to evaluate treatment effects in controlled trials.

## Methods

The objective of the study was to develop a reliable and valid measure of CHIKA disease activity. A methodologic framework for selection of disease activity measures in RA clinical trials was applied to the development of a chikungunya specific disease activity measure. An expert group of rheumatologists, international CHIKA experts and biostatisticians identified component measures for possible inclusion in the composite disease activity score. Because the Disease Activity Score-28 with C-reactive protein (DAS-28) is currently the primary method to assess disease severity for CHIKA patients and is currently used for outcomes in CHIKA trials due to the lack of a CHIKV specific measure [12], this measure was included as a prime candidate for inclusion in a CHIKA focused measure of disease activity. The DAS-28 includes assessment of the number of swollen and tender joints (out of the 28), CRP, and a visual analog global assessment of health. Based on a study by Wells *et al*. [11], a DAS-28 score of less than 2.6 meets criteria for remission, a score greater than or equal to 2.6 and less than 3.1 indicates low activity; a score greater than or equal to 3.1 and <5.1 indicates moderate activity and a score 5.1 or more indicates high activity. In addition to the DAS-28, additional measures for possible inclusion in a new CHIKDAS score were selected by the expert group for further evaluation if there was a good consensus that the measures demonstrated face validity, were relevant to clinical disease and sampled multiple correlating domains of chikungunya arthritis disease.

These potential additional measures were evaluated using prospectively collected longitudinal observational clinical trial data from CHIKA patients in Colombia. Using this dataset, we assessed criterion validity by determining which, if any, of these measures improved prediction beyond the DAS-28 of patient reported outcomes of disability, mobility, pain and physical and mental quality of life. These outcome measures were selected because they represent clinically relevant patient outcomes. For example, disability as measured by the Health Assessment Questionnaire (HAQ) Disability Index [10] has been correlated with mortality rates, progression of aging, and health care resource utilization in diverse pathologies. Furthermore, measurement of pain is of specific importance to quantify CHIKA impact as it is associated with multiple domains of QoL [8]. Given the conceptual overlap and self-reported nature of the 5 outcome measures, the possibility of a largely unidimensional structure to the data was explored through Pearson correlations and internal consistency reliability of a single “composite outcome” score.

Lastly, we used linear regression to select variables independently predicting patient reported outcome and used the resulting regression weights to construct CHIK-DAS scores. These scores were further transformed to T-Scores to facilitate their use in clinical practice. Additionally, tentative cut points on the T-scores were used to allow mapping onto disability severity categories widely used with the HAQ.

### Colombian clinical CHIKA cohort

Colombian participants with clinically confirmed CHIKV infection were enrolled as part of a prospective cohort. CHIKV diagnosis was serologically confirmed via IgG antibody immunofluorescence. A baseline in person history and physical was conducted to ascertain demographic characteristics, exposure history, and arthritis signs and symptoms. The race was self-reported with an open-ended question. Blood samples were collected to determine levels of C-reactive protein.

### Setting and participants

Participants were recruited from Magdalena and Atlántico Departments, Colombia, where a local chikungunya epidemic began in 2014. In Colombia, the first laboratory-confirmed autochthonous cases of CHIKV infection were reported in September 2014. From 2014 to 2016, 19,435 chikungunya cases were reported in Colombia [13]. Data from participants over the age of 18 with a history of CHIKV infection were analyzed. As per the Colombian Institute of Health, a clinically confirmed case of CHIKV infection is defined as a fever of >38°C, severe joint pain or arthritis, and the acute onset of erythema multiforme with symptoms not explained by other medical conditions. In addition, these individuals must reside in or have visited a municipality where evidence of chikungunya virus transmission is present or have traveled within 30 km of confirmed viral circulation. All suspected CHIKF cases were laboratory confirmed for the purposes of this study with CHIKV IgG immunofluorescence. Participants were excluded if they did not have a blood sample collected or CHIKV IgG immunofluorescence was negative. The serologically confirmed chikungunya cases have been followed since their infection with CHIKV as part of a study entitled, Chikungunya Arthritis Mechanisms in the Americas (CAMA) in order to characterize arthritis progression. A total of 158 patients provided data for the current analysis. Participants had 1–2 study visits depending on their availability. They were evaluated in 2019 and/or 2021. No study visits were performed in 2020 due to the COVID-19 pandemic. 74 participants were evaluated in 2019, and 84 participants were evaluated in 2021. Participants evaluated in 2019 and 2021 came from the same region and have the same demographic profile. The DAS-28, stiffness, disability, mental and physical quality of life, mobility, pain, and ankle tenderness/swelling were assessed at both timepoints. Assessment of finger and foot joint tenderness and swelling was added in 2021.

### Procedures

Blood was obtained by venipuncture into K2EDTA vacutainers. The blood samples were centrifuged at room temperature (18–25°C) in a horizontal rotor for 20 minutes at 1,500 relative centrifugal force. Plasma was removed from the blood collection tubes and frozen at −80°C until analyzed.

All patients were assigned a unique patient identification number, which was used in the database and for labeling of patient samples. All patient data were free of personal identifiers and were stored in the REDCap database at The George Washington University.

An immunofluorescence-based assay was used to determine the presence or absence of anti-chikungunya virus IgG antibodies in enrolled patients (EUROIMMUN, Germany). Plasma samples were used to coat slides fitted with biochips containing chikungunya positive and negative cells. If IgG was present in the sample, the antibody would react with positive cells and fluoresce. Slides were read using the 488 nm excitation filter on a Biotek Lionheart LX fluorescent microscope. Measurement of C-reactive protein was performed using the AccuBind high sensitivity C-reactive protein ELISA kit as per the manufacturer’s instructions. Anti-cyclic citrullinated peptide (CCP) antibody ELISA (EUROIMMUN, Germany) was performed as per the manufacturer’s instructions. Microtiter plates coated with CCP were incubated for 60 min with plasma samples (1:101 dilution) before washing. Anti-human IgG peroxidase-conjugate was added to each well, followed by washing. For visualization, 3,3,5,5 tetramethylbenzidine enzyme substrate was added. Optical density (OD) was determined at 450 nm (reference wavelength 620 nm). All procedures were carried out at room temperature. The cut-off value was 5 relative units (RU)/ml.

The study complies with the Declaration of Helsinki. The study protocol was approved by The George Washington University Institutional Review Board (Protocol: Colombian Arboviral Surveillance Protocol IRB #121611, GWU IRB, Washington, D.C., USA (FWA00005945) and the Clínica de la Costa IRB, Colombia (FWA IORG0008529)). Research on human subjects was conducted in compliance with regulations relating to the protection of human subjects and adheres to principles identified in the Belmont Report (1979). All data collection and research on human subjects for this publication were conducted under an IRB-approved protocol. All participants were adults and provided written informed consent during an in-person interview.

### Statistical analysis

Bivariate associations among variables were examined by scatterplots and Pearson correlations. Because 3 of the 6 potential CHIK-DAS components were only collected for 84 of the 158 participants, a multiple imputation technique using SAS software (version 9.4) [14] was used to incorporate all available data to estimate Pearson correlations and regression models. Because the analysis variables of interest were all continuous and not substantially skewed, the MCMC approach for multiple imputation was applied to a subset of the database containing all the candidate components and outcome measures. Imputation software calculations were based on initial estates using the EM algorithm, the Jeffreys prior, 200 burn-in iterations, and the creation of 100 imputation datasets. Pearson correlations for variables containing missing data were estimated by averaging estimates for the 100 datasets after applying the Fisher’s z transformation and then back-transforming that average.

Selection of independent correlates of each of the composite outcomes was through multivariable linear regression estimated through “Rubin’s rule” for multiple imputation. A composite outcome score was first constructed by averaging the 5 patient outcome measures after converting them to z-scores and adding this score to the imputation datasets. The backward selection method was used, which started with six predictors. At each step the variable with the highest p-value was removed and the model re-estimated until all remaining predictors met a criterion of p >0.05. This backward selection approach has been found to work well with multiple imputation [15]. Multicollinearity was assessed through the variance inflation factor (VIF) statistic as calculated on the first random imputation dataset. After consensus determination of the final components to include in the CHIK-DAS, scores were calculated using the unstandardized regression equations coefficients predicting the composite outcome. The final formula subtracted the predicted scores from 50 and divided by the standard deviation/10 to produce T-scores.

Although exact power cannot readily be calculated for models employing multiple imputation and backward selection, the following provides a rough guide to expected power for the regression-based selection approach used here: If the effective sample size is 121 (estimated to account for missing data by averaging n=84 and n=158), and five variables predict the outcome with R^2^ = 0.20, there would be 80% power (using SAS Proc Power) to detect an R^2^ increase of 0.05 in connection with a sixth predictor (alpha=0.05).

## Results

### Baseline characteristics of the study population

The baseline characteristics of the study population are shown in Table 1. The participants were predominantly Mestizo female subjects (mean age 49 ± 16) with at least secondary school level of education. The average duration of time since chikungunya infection was approximately 5 years. One participant was anti-CCP antibody positive. C-reactive protein varied widely.

### Measures

An expert group identified component measures for possible inclusion in the composite disease activity score shown in Table 2. Out of 12 measures considered, 8 were selected as candidates for inclusion in the CHIK-DAS. This was based on consensus among 9 clinicians (AYC, HW, AS, JJJ, KA, GS, LM, AC, and GSF) with extensive experience in diagnosing and treating patients with CHIKA. These measures were selected for the frequency in CHIKA, correlation with radiographic disease, correlation with perceived disease severity, and ease of collection in a clinical or research setting. Specifically, ankles swelling, metatarsal phalangeal swelling, and tarsal midfoot tenderness and swelling were considered for inclusion as CHIK-DAS candidates but were omitted due to low frequency of these events in CHIKA. Furthermore, toe proximal interphalangeal joint tenderness and swelling were omitted due to reported difficulties with accurate and timely clinical assessment reported by the expert group’s rheumatologists.

Patient reported outcomes were selected to provide reliable and widely used measures of disability, pain, physical and mental quality of life, and mobility (Table 3). Correlations among the patient reported outcomes are shown in Supplementary File Table 1. These ranged from 0.25 to 0.84 in absolute value. Given the high correlations, the five measures were averaged into a single composite outcome score after being converted to z-scores. This outcome measure was used to determine which internal component measures might be of value for inclusion in the CHIK-DAS.

The mean values of component measures considered for inclusion in the CHIK-DAS and external measures of patient reported outcomes for the study populations are also shown in Table 1. Notably, moderate stiffness was reported. The DAS-28 indicated moderate disease activity among the participants. A tender ankle joint, finger distal interphalangeal joint and foot metatarsophalangeal joint were present in most participants. Mean disability scores were mild to moderate. Median visual analog pain scores were moderate. The mental and physical quality of life and mobility scores were somewhat below average [16].

Correlations among the six candidate component measures are shown in Supplementary File Table 2. The strongest correlations are between the Finger DIP Tender Joint Count and the Foot MTF Tender Joint Count (r=0.65), as well as between the latter and the DAS-28 (r=0.50), and the DAS-28 with the Stiffness score. All other correlations among outcomes were at r=0.44 or lower. Correlations among the five outcome measures were all high, ranging in absolute value from r=0.51 to r=0.86 (Supplementary File Table 2). The estimated coefficient alpha internal consistency was 0.91, based on summing the z-scores (SD forced to 1.0) for the five outcome measures.

Correlations between the CHIK-DAS Candidate components and the composite outcome varied widely (Table 4), with DAS-28 and stiffness having the highest correlations. Finger DIP Swollen Joint Count was the weakest (r=0.16).

### Regression model selection of independent correlates of outcomes

Table 5 shows the regression coefficients for 3 models predicting composite outcome. The simplest model includes the DAS-28 score by itself as a predictor. Here the coefficient is statistically significant, with a model R^2^ of 0.44. A second “all candidate components” model includes DAS-28 plus 5 additional candidate components for a CHIK-DAS score. Of these, only the DAS-28 and stiffness scores reached statistical significance. Confidence intervals for DAS-28 and Finger DIP Swollen Joint Count are relatively wide in that model, the former may be a consequence of moderately elevated multicollinearity (VIF=2.1). Finally, a backward selection approach was applied to the “all candidate components” model. The resulting model included the DAS-28, the stiffness score, and the ankle tender joint count. The addition of the stiffness score and ankle tender joint count resulted in an increase in the R^2^ to 0.54

### Expert consensus on chikungunya components to include in disease activity score

**Figure 1.**
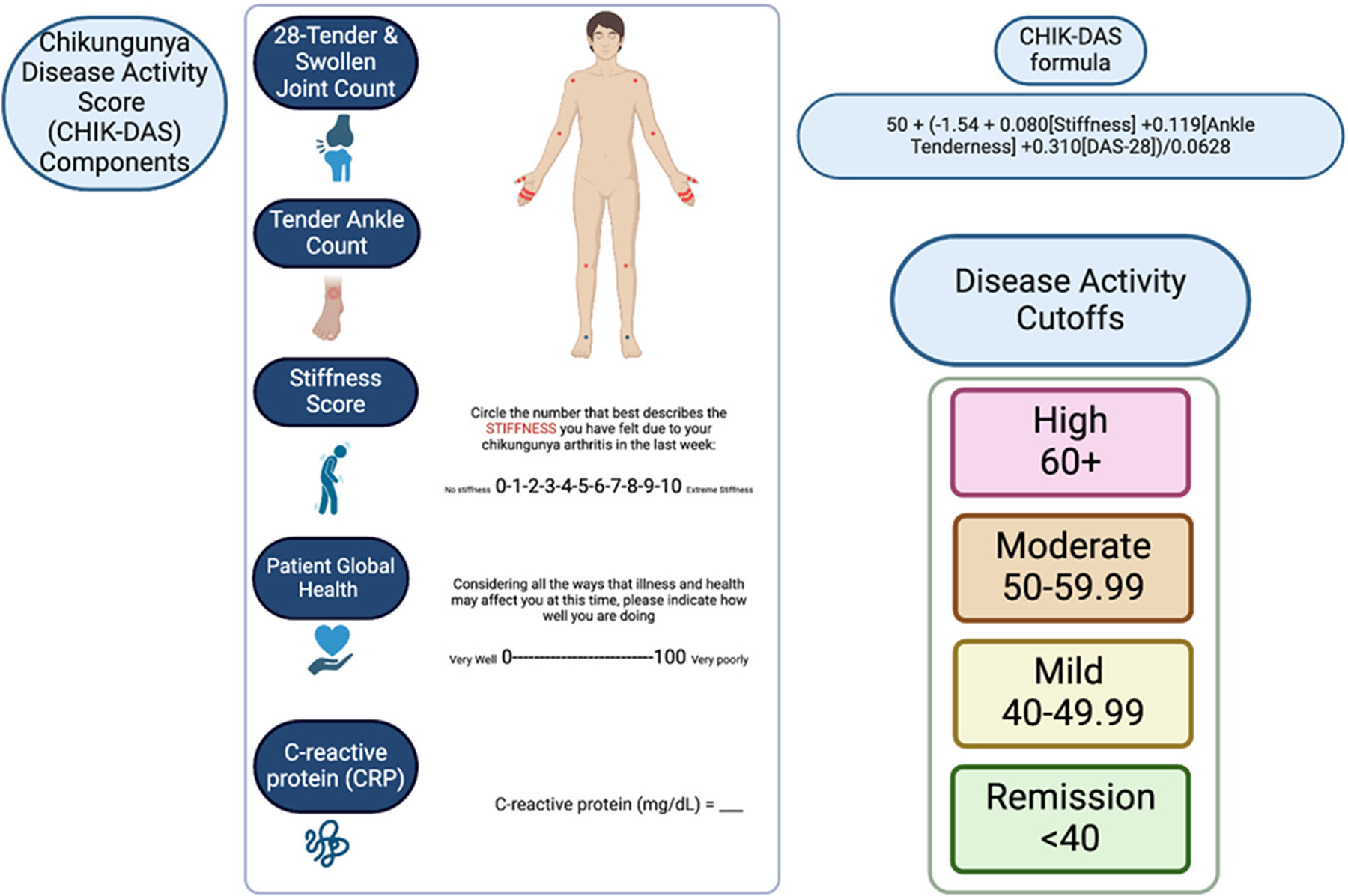
Chikungunya Disease Activity Score (CHIK-DAS). The items include the DAS-28 with CRP that includes a patient global health measure, ankle tenderness and a stiffness score. The 28 joints included in the DAS-28 are the bilateral shoulders, elbows, wrists, metacarpophalangeal, proximal interphalangeal and knees. The formula and scoring for the disease activity cutoffs are shown. Created with BioRender.com.

An expert group of four rheumatologists, nine chikungunya arthritis experts from the United States, Colombia, Brazil, and France and two biostatisticians decided on the final components of the CHIK-DAS. Experts were selected based on clinical and research expertise with chikungunya arthritis. Conflicts of interest were disclosed among the group including conflicts with regard to Valneva, Abbvie, Pfizer, Johnson & Johnson, Briston Myers Squibb, Evotec, Eli Lilly and EUROIMMUN. The final components of the CHIK-DAS were decided considering the findings from the regression selection analyses, ease of use of the scale in clinical practice, and utility for clinical trials. The proposed components of the CHIK-DAS include the DAS-28 and ankle joint tenderness and a stiffness score, as shown in Figure 1.

Based on the consensus components, CHIK-DAS scores were calculated based on the regression model coefficients and standard deviation in the current sample as follows:


CHIK-DASTscore=50+(−1.54+0.080×Stiffness+0.119×AnkleTenderness+0.310×DAS28)/0.0628

Observed range in sample 30.2 to 69.8.

### Mapping the CHIK-DAS to Disability

CHIK-DAS T-scores showed a positive association with HAQ Disability scores. All 27 participants with CHIK-DAS T-scores below 40 had HAQ disability scores in the mild/moderate range (below 1.0), while 62% of those with CHIK-DAS T-scores of 60 or higher were classified as “moderate/severe” or “severe/very severe” on the HAQ (Supplementary File Table 3). Only 4 participants were in the severe/very severe range of the HAQ, suggesting insufficient data in this sample to reliably map CHIK-DAS T-scores to very severe disability.

### Determining cutoffs for disease severity in the CHIK-DAS

Disease activity cutoffs were defined for remission (<40), mild (40–49.99), moderate (50–59.99) and severe (60+) disease. As the CHIKDAS is a T-score, 40 and 60 represent one standard deviation from mean. The cutoffs were determined based on mapping to clinically relevant outcomes including the arthritis disease activity score-28, stiffness, ankle tenderness and disability as shown in Table 6 where mild, moderate and severe outcomes were mapped to mild, moderate and severe CHIK-DAS classification. The remission cutoff corresponds to a DAS-28 score in remission, minimal stiffness, no swollen ankles, and minimal disability. The mild CHIK-DAS group is characterized on average by a mild DAS-28, mild stiffness, a swollen ankle in half the participants, and mild disability. The moderate CHIK-DAS group is characterized on average by moderate CHIK-DAS, moderate stiffness, one swollen ankle and moderate disability. The severe CHIK-DAS group is characterized on average by a severe CHIK-DAS, severe stiffness, 1–2 swollen ankles per participant and severe disability.

## Discussion

### CHIK-DAS: A novel specific metric for measuring disease severity in chikungunya arthritis

We have developed the first disease activity score that is developed and validated for use in CHIKA. The anticipated use could be either in the context of clinical trials or in clinical practice. The basis for most of the measures used in CHIKA has been their value in RA, although our process clearly defined other important components that do not have a crucial role in the DAS-28 [12,17]. For example, DAS-28 prioritizes the joints of the upper limb while CHIK has significant lower limb involvement [12,18,19]. Thus, we determined that the inclusion of ankle joints to a tender joint count improved the performance of the metric. In addition, tender/painful joints are consistently more frequent than swollen joints in this condition [20]. While often lacking frank swelling, tender and painful joints in CHIKA patients with tenosynovitis demonstrate inflammation with musculoskeletal ultrasound power Doppler and greyscale [21] and were an important variable for inclusion. Stiffness was also included given previous studies highlighting its relevance to patient outcomes in several chronic CHIK cohorts (Colombia, Brazil, Guadeloupe) [8,20,22] and correlation with musculoskeletal ultrasound power Doppler and greyscale evidence of inflammation [21]. Therefore, as tenderness/painful joints are consistently more frequent than swollen joints in this condition [20]; distal joints are frequently involved [12,19,20]; and stiffness is associated with outcomes such as disability and mobility [8,20] the CHIK-DAS was developed to reflect the specific disease profile of CHIKA. With the addition of only ankle tenderness and a stiffness measure, the CHIK-DAS demonstrates excellent internal consistency with an estimated coefficient alpha internal consistency of 0.91 and performs better than the DAS-28 alone to capture chikungunya specific arthritis disease severity.

One participant was anti-CCP antibody positive almost eight times the upper limit of normal suggesting that this patient has rheumatoid arthritis. This patient had no history of arthritis prior to CHIKV infection, and his joint pain began with CHIKV infection. This patient was not excluded from the analysis as there is some evidence that suggests that citrullinated vimentin in the synovium due to chikungunya replication may result in anti-CCP antibodies in CHIKVA in cases with a genetic predisposition and may contribute to the development of rheumatoid arthritis [23].

The limitations of the CHIK-DAS with and without CRP include the need for further validation in additional cohorts with longer and shorter durations of arthritis to see how robust the metric is in different populations. This study was validated in a single Colombian Mestizo population and additional diverse cohorts are needed to enhance the generalizability of the tool. For example, examination of how the metric performs in other ethnic groups would be of interest. Additionally, there may be some recall bias regarding the CHIKV infection that was years ago in this cohort highlighting the need for validation in a cohort with recent CHIKV infection. Furthermore, assessment of correlation with radiographic evidence of disease should be investigated in a validation cohort. A backward selection approach was used for selection of the variables for inclusion in the metric. Multiple other approaches for item selection are possible and may provide slight variations in the variables suggested for inclusion. The sample size for this validation cohort was (N=158) for all measures except three (N=84). Linear regression weights for scoring are sample-dependent and will shift somewhat in future versions of the CHIK-DAS as more cohorts are used to validate the scoring system. A stringent p-value of <0.05 for inclusion in the model was used in this study; however future studies may consider the use of less stringent criteria to identify a broader range of components for inclusion. While we included a mapping of CHIK-DAS scores to disability severity descriptions used with the HAQ, empirically derived cut point estimates for this purpose can be unstable and should be seen as rough descriptors.

In conclusion, the CHIK-DAS is a novel measure of chikungunya arthritis disease activity that may be utilized in clinical trials and clinical use to define evidence-based treatments for this neglected disease. As CHIKV arthritis occurs predominantly in underserved regions, having a validated tool to measure chikungunya arthritis disease severity becomes one mechanism to address global health disparities. Further validation in additional cohorts is needed.

## Supplementary Material

JCI-24-211_Supplementary files

## Figures and Tables

**Table 1.** Baseline characteristics of the participants with chikungunya arthritis (N=158).

Characteristic	Mean (SD) or Percent	Minimum, Maximum
**Demographics and Clinical Information**		
Age	48.6 (15.8)	18, 87
Female	80%	
With at least secondary school education (n=157)	63%	
Mestizo Ethnicity	100%	
Duration of time since CHIKV infection (in days)	1,632 (531)	0, 4416
Anti-cyclic citrullinated peptide (CCP) antibody positive	1.20%	
C-Reactive Protein (mg/dl) (n=157)	2.68 (3.76)	0, 24.49
**Component measures evaluated for inclusion in the CHIK-DAS**		
Stiffness score	4.05 (3.16)	0, 10
DAS-28	3.63 (1.31)	0.96, 6.65
Ankle Tender Joint Count	0.78 (0.90)	0, 2
Finger Distal Interphalangeal Tender Joint Count (n=84)	0.81 (1.86)	0, 8
Finger Distal Interphalangeal Swollen Joint Count (n=84)	0.17 (0.67)	0, 5
Foot Metatarsophalangeal Tender Joint Count (n=84)	1.24 (3.46)	0, 18
**External measures of patient reported outcomes**		
Disability *Health Assessment Questionnaire Disability Index*	0.74 (0.55)	0, 2.88
Pain *Visual analog scale*	6.07 (2.78)	0, 10
Physical Quality of Life *PROMIS-29 Physical Health Domains*	45.8 (8.5)	25.2, 58.7
Mental Quality of Life *PROMIS-29 Mental Health Domains*	47.3 (8.0)	29.7, 65.2
Mobility *PROMIS Mobility Measure*	43.1 (9.6)	15.5, 61.9

**Table 2.** Internal component measures evaluated for inclusion in the Chikungunya Disease Activity Score (CHIK-DAS).

Measure	# of Items	Scoring	Rationale
Stiffness score	1	A visual analog scale ranging from 0–10 adapted from the Outcome Measures in Rheumatology (OMERACT) rheumatoid arthritis flare questionnaire where the participant is asked to “Circle the number that best describes the STIFFNESS you have felt due to your chikungunya arthritis in the last week.”	Stiffness in the absence of swelling has been associated with disability and decreased quality of life (QoL) in CHIKA [9].
DAS-28	28	Calculated score including tender and swollen joints including glenohumeral joints (0–2), elbows (0–2), wrists (0–2), metacarpal phalangeal joints (0–10), finger proximal interphalangeal joints (0–10), and knees (0–2), patient global health (0–100) and C-reactive protein.	The DAS-28 thus far has been the standard measure applied for CHIKA trials due to the lack of a CHIKV specific measure [11].
Ankle Tender Joint Count	2	Sum of tender ankle joints (0–2)	The smaller joints of the fingers, ankles and feet are among the most commonly affected joints in CHIKA [11].
Finger Distal Interphalangeal Tender Joint Count	8	Sum of tender distal interphalangeal joints (0–8)	
Finger Distal Interphalangeal Swollen Joint Count	8	Sum of swollen distal interphalangeal joints (0–8)	
Foot Metatarsophalangeal Tender Joint Count	10	Sum of tender metatarsophalangeal joints (0–10)	

**Table 3.** External measures of patient reported outcomes of interest in chikungunya arthritis used to validate possible internal component measures of the Chikungunya Disease Activity Score (CHIK-DAS).

Measure	# of Items	Scoring
**Disability** *Health Assessment Questionnaire Disability Index*	20	For each item, there is a four-level difficulty scale that is scored from 0 to 3, representing normal (no difficulty) (0), some difficulty (1), much difficulty (2), and unable to do (3). There are 20 questions in eight categories of functioning - dressing, rising, eating, walking, hygiene, reach, grip, and usual activities. The value of the HAQ index can be interpreted in terms of three categories: mild difficulties to moderate disability (0–1), moderate to severe disability (1–2), and severe to very severe disability (2–3).
**Pain** *Visual analog scale*	1	Pain was assessed with a visual analog scale ranging from 0–10.
**Physical Quality of Life** PROMIS-29* *Physical Health Domains*	12	PROMIS-29 physical health domains include Physical Function, Fatigue, and Sleep Disturbance with 4 questions for each domain. Each question has five response options ranging in value from one to five that are summed for a total raw score and a standardized T-score is then calculated in which 50 is the mean of a relevant reference population and 10 is the standard deviation of that population.
**Mental Quality of Life** *PROMIS-29 Mental Health Domains*	8	PROMIS-29 mental health domains include Depression and Anxiety with 4 questions for each domain. Each question has five response options ranging in value from one to five that are summed for a total score and a standardized T-score is then calculated in which 50 is the mean of a relevant reference population and 10 is the standard deviation of that population.
**Mobility** *PROMIS Mobility Measure*	4	PROMIS Mobility Measure has 4 questions. Each question has five response options ranging in value from one to five that are summed for a total raw score and a standardized T-score is then calculated in which 50 is the mean of a relevant reference population and 10 is the standard deviation of that population. Loss of mobility is graded as mild (40–50), moderate (30–40) and severe (0–30).

* Abbreviation: Patient-Reported Outcomes Measurement Information System (PROMIS)

**Table 4.** Correlations between CHIK-DAS candidate components and composite outcome.

Component	Composite Outcome
DAS-28	0.68***
Ankle Tender Joint Count	0.39***
Finger DIP Tender Joint Count	0.31**
Finger DIP Swollen Joint Count	0.16
Stiffness score	0.56***
Foot MTF Tender Joint Count	0.28**

* p< 0.05;

** p< 0.01;

*** p<0.001

**Table 5.** Linear regression coefficients for models predicting composite outcome from candidate components.

Component	Model		
	DAS-28 Only	All candidate components	Final Selected
DAS-28	0.44*** (0.37, 0.52)	0.35*** (0.23, 0.45)	0.31*** (0.44, 0.40)
Stiffness Score	--	0.086*** (0.05, 0.12)	0.081*** (0.05, 0.11)
Ankle Tender Joint Count	--	0.099 (−0.03, 0.22)	0.121* (0.04, 0.23)
Finger DIP Tender Joint Count	--	−0.027 (−0.12, 0.07)	--
Finger DIP Swollen Joint Count	--	−0.15 (−0.36, 0.07)	--
Foot MTF Tender Joint Count	--	0.014 (−0.04, 0.07)	--

* p< 0.05;

*** p<0.001

Note. N=158 for DAS-28, Stiffness Score, and Ankle Tender Joint Count; N=84 for remaining components. Estimates utilized all available data through multiple imputation for the “All candidate components model”.

**Table 6.** Observed Component and Disability Means (SD) for proposed CHIK-DAS named levels.

Scale (min, max)	Remission (<40) [n=27]	Mild (40–49.99) [n=44]	Moderate (50–59.99) [n=58]	Severe 60+) [n=29]
DAS-28 (1, 6.7)	1.6 (0.7)	3.2 (0.7)	4.0 (0.6)	5.3 (0.6)
Stiffness (0, 10)	0.3 (0.7)	2.4 (2.6)	5.4 (2.2)	7.4 (1.4)
Ankle Tenderness (0, 2.0)	0.0 (0.0)	0.5 (0.8)	0.9 (0.9)	1.5 (0.8)
HAQ (0, 2.9)	0.2 (0.3)	0.6 (0.4)	0.9 (0.5)	1.2 (0.6)

## Data Availability

The dataset supporting the conclusions of this article is included within Supplementary File 4 Dataset.
